# Alterations of Glymphatic System Before and After Shunt Surgery in Patients With Idiopathic Normal Pressure Hydrocephalus: A Longitudinal Study

**DOI:** 10.1111/ene.70200

**Published:** 2025-05-14

**Authors:** Yifeng Yang, Meijing Yan, Xiao Liu, Shihong Li, Guangwu Lin

**Affiliations:** ^1^ Department of Radiology Huadong Hospital, Fudan University Shanghai China

**Keywords:** cognitive function, diffusion tensor imaging along the perivascular space (DTI‐ALPS), glymphatic function, idiopathic normal pressure hydrocephalus, longitudinal study

## Abstract

**Aims:**

This study aimed to assess the glymphatic dysfunction in idiopathic normal pressure hydrocephalus (iNPH) patients and its recovery post‐shunt surgery using diffusion tensor image analysis along perivascular spaces (DTI‐ALPS).

**Methods:**

Thirty‐five iNPH patients and forty healthy controls (HC) underwent MRI scans and neuropsychological assessments at baseline. A follow‐up study, conducted three months post‐shunt surgery, included fifteen iNPH patients. The DTI‐ALPS index was calculated to assess the glymphatic system status. Group differences were evaluated using the Mann–Whitney *U* test, while the paired Wilcoxon signed‐rank test was employed to compare pre‐operative and post‐operative ALPS indices. Multiple linear regression was utilized to analyze the association between changes in the ALPS index (ΔALPS) and alterations in clinical scores.

**Results:**

Baseline examinations disclosed iNPH patients had a lower ALPS index than HC (*p* < 0.0001). We found a significantly increased ALPS index at 3 months after surgery compared to baseline (*p* < 0.0001). Positive correlations between theΔALPS and the increments of MMSE score (ΔMMSE) were found in all iNPH patients. Baseline age and ΔALPS emerged as significant predictors of ΔMMSE, with the model explaining 68.13% of the variance (*R* ^2^ = 0.6813).

**Conclusion:**

Glymphatic function in iNPH was enhanced following shunt surgery, which positively impacted cognitive recovery. The DTI‐ALPS index may serve as a useful predictor of shunting efficacy in iNPH patients.

## 1 Introduction

Cerebrospinal fluid (CSF) shunt surgery is an effective treatment for idiopathic normal pressure hydrocephalus (iNPH), leading to significant improvements in patient symptoms and prognosis. By draining excess CSF, the surgery restores abnormal CSF pulsation and compensates for insufficient CSF absorption [1, 2]. Meanwhile, the ventricular decompression achieved through shunt surgery restores impaired cerebral blood flow and normalizes subsequent pathophysiological changes [3, 4]. However, the exact mechanisms underlying these beneficial effects remain incompletely understood.

The recently discovered brain glymphatic system facilitates the flow of CSF along perivascular spaces (PVS), aiding in the clearance of excess metabolic waste from the central nervous system (CNS) [5]. Dysfunction in the glymphatic system can lead to the accumulation of toxic proteins, associated with various physiological conditions and neurological disorders, including aging [6], sleep [7], Alzheimer's disease, and Parkinson's disease [8, 9, 10]. Impaired glymphatic function is noted in iNPH patients, with MRI studies showing delayed CSF tracer clearance in these individuals, particularly in the subarachnoid space [11], entorhinal cortex [12, 13], and visual pathways [14]. Research by Eide et al. revealed that gadobutrol was absorbed from the CSF by the choroid plexus, but this process was delayed in iNPH patients [12], with both Sperre et al. and Eide et al. reporting gadobutrol reflux and enrichment in the ventricles [15, 16].

The innovative Diffusion Tensor Image Analysis along the Perivascular Space (DTI‐ALPS) method, a non‐invasive imaging technique, has been developed to assess the global glymphatic system by measuring perivascular water flow around the ventricles [17]. It has been widely used in neurological disorders, revealing impaired glymphatic function [9, 10, 18, 19]. The research identified a reduced ALPS index in iNPH patients [20]. Non‐responders to shunt surgery showed a significantly lower index than responders, indicating that poor postoperative outcomes might be associated with severely impaired glymphatic activity [21]. This underscores the importance of glymphatic health in disease progression and treatment success. However, changes in glymphatic function in iNPH patients before and after shunt surgery are still not well understood.

The brain's glymphatic activity is crucial for cognitive function. Disrupted glymphatic systems hinder the clearance of neurotoxic substances, affecting neuronal function and causing neuroinflammation [22, 23, 24]. Animal studies indicate that improving glymphatic clearance can enhance cognition [25, 26, 27]. A study on carotid stenosis patients showed that increased DTI‐ALPS index post‐stenting correlated with better MMSE scores improvement, suggesting early interstitial fluid flow improvements can boost long‐term cognitive outcomes [28]. Therefore, we hypothesize that the restoration of CSF dynamics following shunt surgery in iNPH patients might also promote glymphatic flow, potentially as a mechanism for cognitive function recovery.

This study used the DTI‐ALPS index to assess glymphatic flow changes post‐surgery in iNPH patients. Additionally, we aimed to investigate the potential correlation between post‐surgical improvements in glymphatic flow and alterations in cognitive function to understand the potential role of glymphatic function in the pathogenesis and recovery of iNPH.

## 2 Materials and Methods

This research received approval from the institutional review board at Huadong Hospital, affiliated with Fudan University (approval number: 2017K027). All participants provided written informed consent.

### 2.1 Study Participants

**FIGURE 1:**
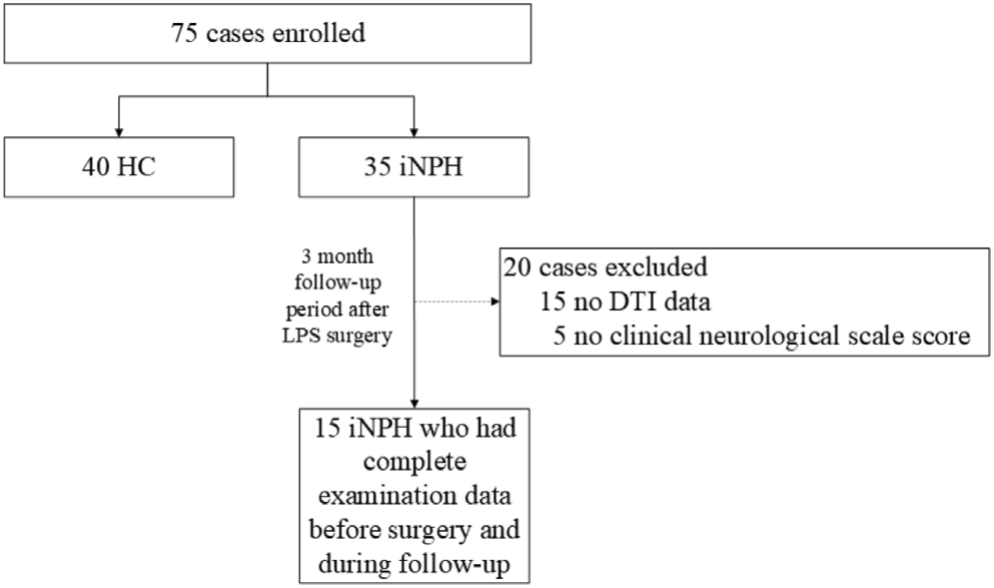
Flow chart of participator inclusion. HC, health control; iNPH, idiopathic normal pressure hydrocephalus; LPS, Lumbar‐peritoneal shunt surgery.

Between January 2014 and December 2023, 35 iNPH patients and 40 healthy controls (HC) underwent MRI scans and neuropsychological assessments at baseline. The diagnosis of iNPH was conducted in accordance with the protocols outlined in the third edition of the guidelines [29]. Detailed inclusion and exclusion criteria are available in the Appendix S1. A follow‐up study three months post‐shunt surgery included 15 iNPH patients for whom comprehensive clinical data and MRI imaging were available (Figure 1).

### 2.2 Clinical Symptoms Assessments

All iNPH patients underwent detailed clinical evaluations, which included the Idiopathic Normal Pressure Hydrocephalus Grading Scale (INPHGS), the Mini‐Mental State Examination (MMSE), and the Timed Up and Go test (TUG).

### 2.3 MRI Protocol

All iNPH patients underwent MRI scans with the same protocol before surgery and three months after LP shunt surgery, while HC participants received only one MRI scan. All MRI scans were conducted using a 3T MRI system (Prisma, Siemens). The scanning parameters can be found in Supporting Information.

### 2.4 MRI Data Processing

#### 2.4.1 Ventriculomegaly Measurements

**FIGURE 2:**
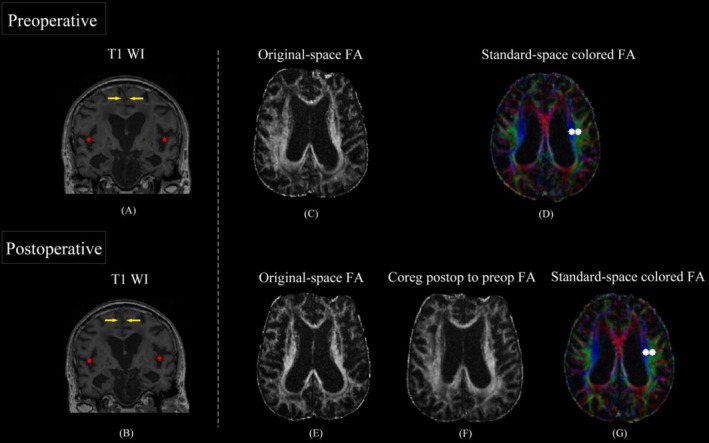
The image processing of pre‐ and post‐operative 3‐month follow‐up T1‐weighted images (T1 WI) and diffusion tensor imaging (DTI) images. (A) and (B) Pre‐ and post‐operative T1 WI demonstrated the DESH sign: The subarachnoid space is narrowed on the high convexity/midline of the brain (yellow arrow), and the bilateral sylvian fissures are significantly widened (red star). (C) Preoperative FA map in original space. (D) Preoperative FA map in standard space. The affine and non‐linear transformations were applied to register each individual FA map to the standard space of the JHU ICBM‐DTI‐81 template. Two 3‐mm‐diameter spherical ROIs were placed on left projection fibers and association fibers along the body of left lateral ventricle, respectively. (E) Postoperative FA map in original space. (F) Postoperative FA map was aligned with preoperative FA map. The linear registration (FLIRT) method was applied to register the postoperative FA map to the preoperative map, and the resulting transformation matrix was applied to the postoperative tensor maps for better within‐subject alignment. (G) Postoperative FA map in standard space. The transformation matrix aligning the preoperative FA map to standard space was applied on the registered postoperative FA images for spatial consistency. The same preoperative ROIs were positioned in the same place in the postoperative maps to calculate the postoperative ALPS index. For subjects in whom postoperative ROIs were displaced, both experts performed manual fine‐tuning adjustments independently.

The measure of ventricular dilatation, Evans index (EI), was manually measured by radiologists (who were unaware of the final diagnosis) on T1‐weighted images. EI is defined as the ratio between the maximum width of the lateral ventricles' frontal horns on axial slices and the maximum width of the skull. In addition, features of disproportionately enlarged subarachnoid space hydrocephalus (DESH) were consistently observed in both pre‐operative and post‐operative T1‐weighted images (T1 WI) of all patients with iNPH, as shown in Figure 2A,B.

#### 2.4.2 Ventricular Volume Measurements

The ventricular volume and estimated intracranial volume (ICV) were obtained for each subject using T1 WI in FreeSurfer (http://surfer.nmr.mgh.harvard.edu/fswiki, version 7.2.0) with the recon‐all pipeline. The ventricular segmentations were visually inspected and manually corrected if necessary by a trained radiologist. The final ventricle volume was normalized as a ratio of the ICV.

#### 2.4.3 DTI‐ALPS Processing and Analysis

Preprocessing of DTI data was performed with FSL (http://www.fmrib.ox.ac.uk/fsl, version 6.0). The main steps included skull stripping, motion correction, eddy current distortion correction, and diffusion metric images calculation. The computation of ALPS‐index requires the identification of the projection neural fibers (proj) and association neural fibers (assoc), given that the trajectories of them are orthogonal to the PVS *x*‐axis. To ensure the stability of the ALPS index outcomes, we used the ICBM DTI‐81 Atlas to assist in localizing the positions of the two neural fibers [20]. Specifically, we registered each subject's fractional anisotropy (FA) map to the JHU ICBM‐DTI‐81 template. The same transformation matrix was then applied to the Tensor maps. From the diffusion Tensor maps transformed into standard space, diffusivity maps in each direction were extracted along the *x*‐ (*D*_*x*_), *y*‐ (*D*_*y*_), and *z*‐axes (*D*_*z*_). Next, according to the JHU‐ICBM‐DTI‐81 white matter labeling atlas, we manually established two spherical regions of interest (ROIs) with a 3 mm diameter within the periventricular areas of the projection and association fibers (Figure 2C,D). All the measurements were performed in the left hemisphere. The locations of the ROIs were verified through the color‐coded FA maps for each patient. Finally, the diffusivities in the *D*_*x*_, *D*_*y*_, and *D*_*z*_ directions for each ROI were automatically measured. The ALPS index was calculated as previously described by Taoka et al. [17].

$$\text{ALPS}-\text{index}=\frac{\text{mean}\;\left(D_{\text{xproj}},D_{\text{xassac}}\right)}{\text{mean}\;\left(D_{\text{yproj}},D_{\text{zassac}}\right)}$$

where *D*_*xproj*_ and *D*_*xassoc*_ represent the diffusivities of the projection fiber and association fibers along the *x*‐axis direction (*D*_*x*_), while *D*_*yproj*_ is the diffusivity of projection fiber on *D*_*y*_ map, and *D*_*zassoc*_ is the diffusivity of the association fibers on *D*_*z*_ map.

The postoperative DTI images were preprocessed like the preoperative ones for consistency. We applied linear registration (FLIRT) to register the postoperative FA images to the preoperative ones. Next, the generated transformation matrix was applied to the postoperative tensor maps to improve within‐subject alignment. The matrix, which aligned the preoperative FA map to standard space, was also applied to the registered postoperative Tensor images to ensure the spatial position matching of images (Figure 2E,F). Finally, the same preoperative ROIs were used to calculate the postoperative ALPS index (Figure 2G). The change rate in the ALPS index after shunt surgery in iNPH patients was quantified as ΔALPS = (postoperative − preoperative)/preoperative ALPS.

Two experienced radiologists preprocessed the MRI data for all subjects. Then, the measurements of EI index, standardized ventricle volume, and ALPS‐index obtained by the two radiologists were averaged to yield the final results for each individual.

### 2.5 Statistical Analysis

All analyses were conducted using SPSS software (version 26.0). The non‐parametric Mann–Whitney *U* test was employed to compare differences between the iNPH and HC groups. A general linear model (GLM) regression analysis using the ALPS index as the dependent variable and the group (iNPH or HC) as the independent variable, with age and sex as covariates. A partial correlation analysis, adjusting for sex and age, was used to explore the relationship between the preoperative baseline ALPS index and ventricle volume. The diagnostic performance of the ALPS index for identifying iNPH was assessed through Receiver Operating Characteristic (ROC) curve analysis. The paired Wilcoxon signed‐rank test was applied to evaluate differences in ALPS indices among iNPH patients before and after shunt surgery. Finally, multiple linear regression analysis was performed to explore the association between postoperative changes in the ALPS index and alterations in clinical function. The inter‐observer reliability for EI, ventricle volume, and the ALPS index was determined using the intraclass correlation coefficient (ICC).

## 3 Results

### 3.1 Study Population

Table 1 presents the demographic, clinical, and imaging characteristics of the participants. A total of 35 iNPH patients with positive shunt surgery were recruited (26 males and 9 females, with a median (IQR) age of 71 [68–72] years), alongside 40 healthy controls (16 males and 24 females, with a median age of 66 [range 62.25–69.75] years). The inter‐observer agreements between the two readers were excellent for the EI and ventricle volume (all, ICC > 0.8). Furthermore, both the EI and ventricle volume in iNPH patients were significantly greater than those of HC (*p* < 0.001).

**TABLE 1:** Baseline demographic and clinical features of participants.

	iNPH (*n* = 35)^a^	HC (*n* = 40)^a^	*p* ^b^
Age (years)	71.00 (68.00–72.00)	66.00 (62.25–69.75)	0.018*
Sex			
Female	9 (25.71%)	24 (60.00%)	0.006**
Male	26 (74.29%)	16 (40.00%)	
Clinical assessment			
INPHGs			
Gait	3 (2–3)	/	/
Cognition	2 (2–3)	/	/
Urination	3 (2–3)	/	/
Total	7 (6–9)	/	/
TUG	21.85 ± 6.76	/	/
MMSE	18.93 ± 7.78	/	/
Evans index	0.33 ± 0.039	0.24 ± 0.029	< 0.001***
Ventricular volume/TIV	0.06 ± 0.020	0.013 ± 0.004	< 0.001***

*Note:* Significant differences are marked with *p < 0.05, **p < 0.01 and ***p < 0.001.

^a^ Mean (SD); Median (IQR); *n* (%).

^b^ Wilcoxon rank‐sum test.

### 3.2 Baseline DTI‐ALPS Measurements

**FIGURE 3:**
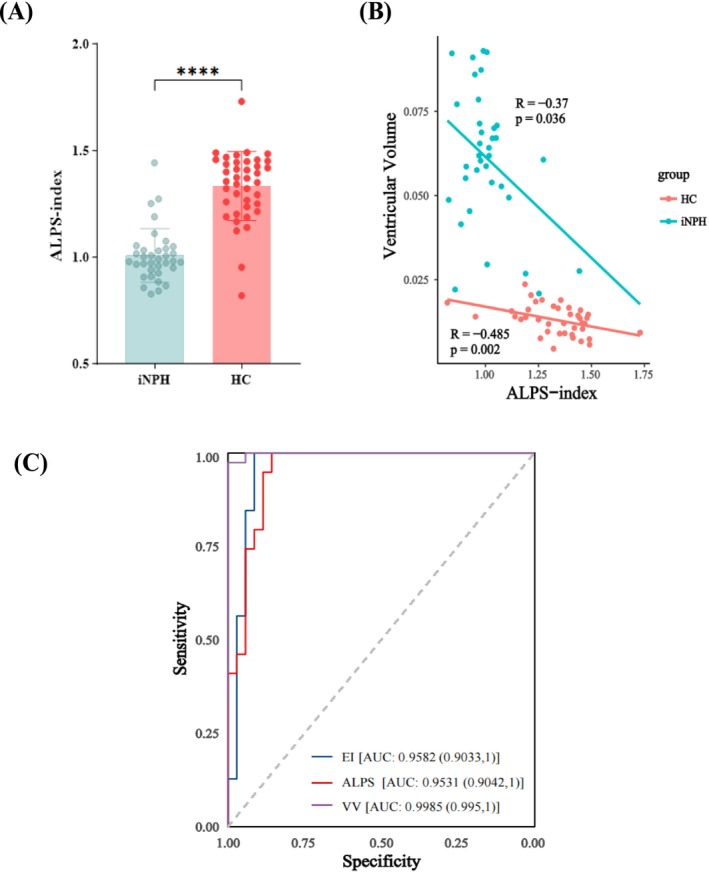
(A) Box plot of ALPS‐index in iNPH and HC groups. The *p* value was obtained from the general linear model (GLM) analysis that adjusted for age and sex. (B) Scatter plot of correlation between ALPS‐index and standardized ventricular volume. A negative correlation was found between them within both iNPH and HC groups. (C) ROC curve between iNPH versus HC. The ability of ALPS to diagnose iNPH is comparable to the EI index. VV, ventricular volume. Significant differences are marked with ****p < 0.0001.

As illustrated in Figure 3A, before undergoing shunt surgery, the average ALPS‐index in iNPH patients was 0.9988 ± 0.1261, significantly lower than that of the HC group (mean ALPS‐index was 1.331 ± 0.1623). After adjusting for age and gender confounders, the correlation coefficient between the ALPS index and ventricle volume in the normal population was *R* = −0.485 (*p* = 0.002); whereas, in the iNPH cohort, it was *R* = −0.37 (*p* = 0.036) (Figure 3B).

The results of the ROC curve analysis, shown in Figure 3C, indicate that the area under the curve (AUC) for the ALPS‐index in identifying iNPH patients was 0.9531 (95% CI: [0.9041, 1]), comparable to the diagnostic performance of the EI index (AUC = 0.9582; 95% CI: [0.9033, 1]), yet lower than that of lateral ventricle volume (AUC = 0.998; 95% CI: [0.995, 1]).

### 3.3 Comparation of DTI‐ALPS Measurements Before and After Shunt Surgery

**FIGURE 4:**
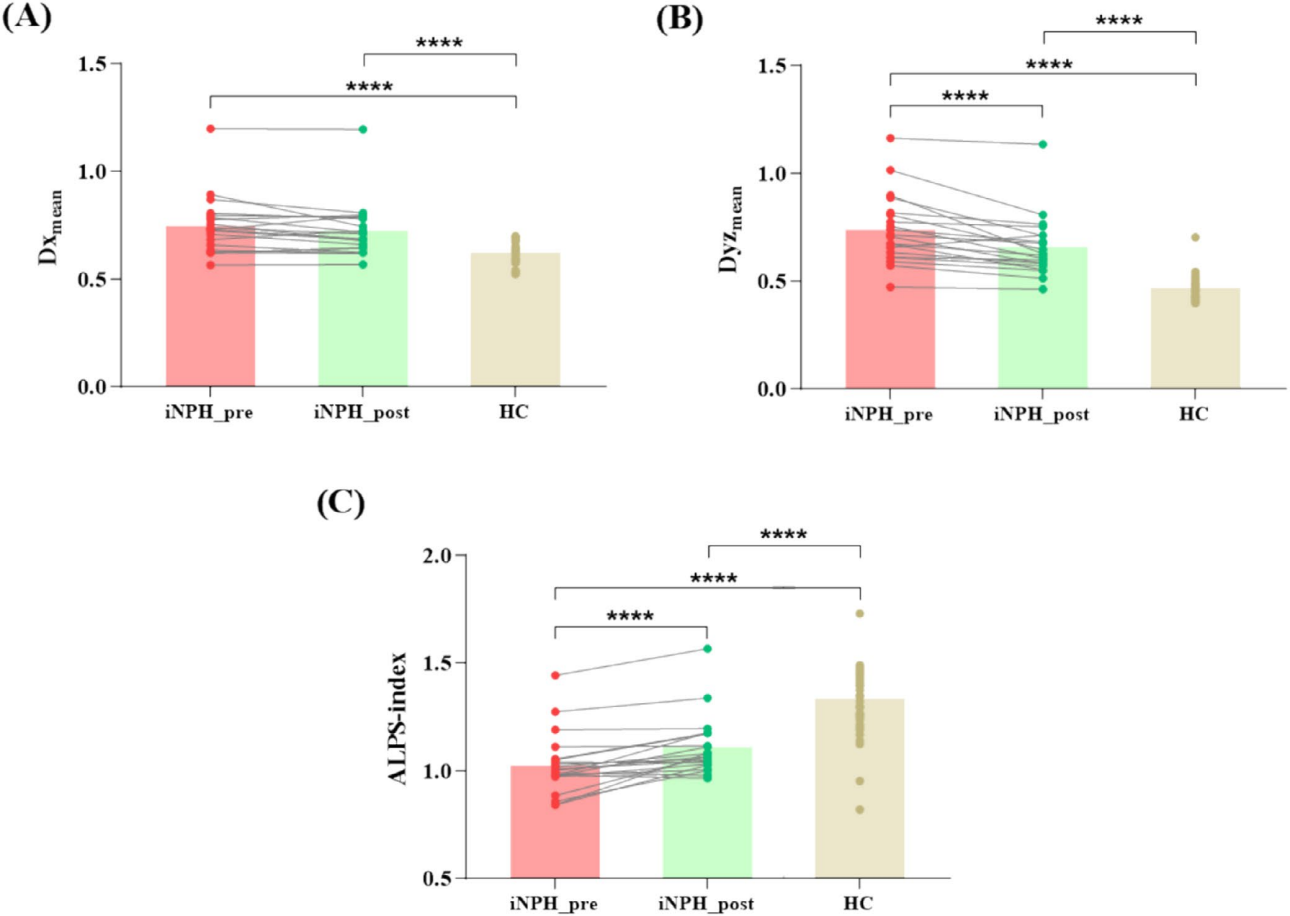
Box plots showed the difference of *D*_*xmean*_, *D*_*yzmean*_, and ALPS index between the pre‐ and post‐shunt surgery in iNPH. Three months after surgery, *D*_*xmean*_ slightly decreased but was not statistically significant, while *D*_*yzmean*_ significantly dropped (*p* < 0.001). The ALPS index significantly rose post‐surgery compared to preoperative levels but was still significantly lower than the HC group (*p* < 0.001).

Table 2 presents a paired comparative analysis of the ALPS index before and after surgery. Here, *D*_*xmean*_ denotes the mean of (*D*_*xproj*_, *D*_*xassoc*_), while *D*_*yzmean*_ represents the average of (*D*_*yproj*_, *D*_*zassoc*_). In comparison with preoperative values, the three‐month postoperative follow‐up exhibited a slight reduction of *D*_*xmean*_ (Figure 4A), though not statistically significant, whereas a significant decline of *D*_*yzmean*_ was noted (*p* < 0.001) (Figure 4B). Overall, the postoperative ALPS index showed a significant increase compared to preoperative levels, yet it remained significantly lower than that of the HC group (*p* < 0.001) (Figure 4C).

**TABLE 2:** *D*_*xmean*_, *D*_*yzmean*_, and ALPS‐index of the iNPH before and after shunt surgery (*n* = 15).

	Pre‐shunt^a^	Post‐shunt^a^	*p* ^b^
*D*_*xmean*_	0.745 ± 0.136	0.724 ± 0.131	0.059
*D*_*yzmean*_	0.738 ± 0.162	0.657 ± 0.142	< 0.001***
ALPS‐index	1.026 ± 0.145	1.116 ± 0.135	< 0.001***

*Note:* *D*_*xmean*_ (×10^−3^ mm^2^/s), mean diffusivity along the *x*‐axis in both projection fiber (*D*_*xproj*_) and association fiber (*D*_*xassoc*_). *D*_*yzmean*_ (×10^−3^ mm^2^/s), mean diffusivity along the *y*‐axis in projection fiber (*D*_*yproj*_) and along the *z*‐axis in association fiber (*D*_*zassoc*_). Significant differences are marked with *p < 0.05, **p < 0.01 and ***p < 0.001.

^a^ Mean (SD); Median (IQR); *n* (%).

^b^ Wilcoxon rank‐sum test.

### 3.4 Correlations Between Clinical Score and ALPS‐Index Increments

The pre‐ and postoperative clinical scores (INPHGS, TUG, MMSE) showed significant improvements after shunt surgery (*p* < 0.001), as detailed in Table S2. Table S3 presents multiple linear regression models linking the ALPS‐index increase (ΔALPS) to changes in clinical scores. Notably, ΔALPS was significantly positively associated with cognitive scores (ΔMMSE) (*β* = 4.74, *p* = 0.001), as detailed in Table 3. The model, including age, gender, and ΔALPS, explained 68.13% of the variance in ΔMMSE.

**TABLE 3:** Multiple linear regression: postoperative MMSE score increment (ΔMMSE) as an outcome variable (*n* = 15).

	Sex		Age		ΔALPS		*R* ^2^
	*β*‐coefficient	*p*	*β*‐coefficient	*p*	*β*‐coefficient	*p*	
Model 1	0.2494	0.2521					0.0995
Model 2			0.0142	0.2907			0.0853
Model 3					2.636	0.0704	0.2301
Model 4	0.2130	0.0617			1.235	0.0213	0.4313
Model 5			0.011	0.0045	4.659	0.0015	0.6181
Model 6	−0.2617	0.167	−0.033	0.0135*	4.74	0.001**	0.6813

*Note:* ΔALPS = (postoperative − preoperative)/preoperative ALPS. Significant differences are marked with *p < 0.05, **p < 0.01 and ***p < 0.001.

Abbreviation: *β*‐coefficient, standardized coefficient beta.

## 4 Discussion

Using DTI‐ALPS, we observed diminished glymphatic function in iNPH patients compared to controls, which also associated with the severity of ventriculomegaly. Subsequently, our longitudinal cohort analysis revealed that iNPH patients exhibited improved glymphatic function three months following shunt surgery, as evidenced by a significant increase in the ALPS index relative to preoperative levels. Additionally, the increase of the DTI‐ALPS index was positively correlated with increased MMSE scores, suggesting that postoperative cognitive function improvements may be linked to the normalization of glymphatic system function. Our study underscores the importance of the glymphatic system in the pathogenesis of iNPH and its prognosis following shunt surgery.

The reduced ALPS index in the iNPH group before shunt surgery indicates impaired glymphatic function and decreased water movement in perivascular spaces, consistent with previous findings [12, 30]. While normal subjects show a nonlinear relationship between age and ALPS index, peaking in their 40s and decreasing thereafter, with a range of 1.2–1.4 for those aged 70–79 [31], our iNPH cohort had a preoperative ALPS index of less than 1.0, below the normal range. This suggests that glymphatic dysfunction in iNPH is disease‐specific, not just age‐related. Factors like AQP 4, sleep state, and vascular pulsations contribute to glymphatic function in iNPH [32, 33]. Previous research has shown that iNPH patients have increased pulsatile intracranial pressure (ICP) and decreased cerebral pulsation absorber index, indicating reduced intracranial compliance [34, 35, 36]. This can limit arterial pulsations, affecting CSF flow and glymphatic clearance. Sleep improves glymphatic clearance, but iNPH patients often suffer from sleep‐disordered breathing (SDB), which can cause intracranial venous hypertension and hinder glymphatic function [37, 38]. Additionally, iNPH patients have lower AQP4 density in perivascular astrocytic endfeet, leading to cerebral edema, cellular dysfunction, and blood–brain barrier issues, further impairing glymphatic flow and increasing Aβaccumulation [23, 39]. Interestingly, even in healthy individuals, there's a notable link between the ALPS index and ventricular volume, suggesting that glymphatic dysfunction directly contributes to ventricular enlargement(Figure 3B). Poor glymphatic function causes waste and fluid buildup, raising intracranial pressure and enlarging ventricles. In iNPH patients, this impaired clearance can worsen the condition, while increased ventricular size can further disrupt glymphatic function. This cycle underscores the importance of glymphatic health to prevent or reduce the impact of ventricular enlargement.

Longitudinal assessments revealed an increase in the ALPS index in iNPH patients post‐shunt surgery compared to pre‐surgery levels, consistent with a small study of six iNPH responders [40]. Specifically, there was a slight decrease in *D*_*xmean*_ and a significant reduction in *D*_*yzmean*_ post‐surgery, indicating a trend toward normalization (see Figure 3). *D*_*yzmean*_ reflects water diffusion in projection and associative fibers, while *D*_*xmean*_ partially captures signals from the PVS. Pre‐surgery, ventricular enlargement in iNPH causes stretching and compression of surrounding white matter and the corpus callosum [41, 42], leading to interstitial edema, axonal loss, and periventricular white matter hyperintensities. Post‐surgery, CSF removal leads to significant absorption of white matter lesions, normalizing diffusion rates in subcortical white matter, especially in periventricular regions and the frontal lobe [43]. Consequently, both *D*_*xmean*_ and *D*_*yzmean*_ values decrease postoperatively, with *D*_*xmean*_ decreasing less than *D*_*yzmean*_. We speculate that this discrepancy indicates enhanced glymphatic fluid flow within the perivascular spaces, likely due to improved CSF circulation. Geir Ringstad and colleagues found that one year after shunt surgery, blood flow direction in the aqueduct had reversed to positive [44]. A. Scollato and associates identified a reduction in the Aqueductal CSF Stroke Volume (ACSV) among patients who responded positively to shunting [45]. The shunt procedure offers an immediate alternative for CSF outflow, enhancing brain expansion and cerebral compliance. Post‐surgery, CSF dynamics improve fluid circulation, blood supply, and glymphatic drainage [46]. Studies also show rapid restoration of cerebral blood flow and perfusion [47, 48], increased cortical metabolism, and changes in CSF biomarkers [49], indicating better glymphatic function. However, we observed that the glymphatic system has not fully normalized. This may be attributable to structural brain changes in iNPH patients affecting the glymphatic system, which would continue to affect the glymphatic system.

Furthermore, we also examined the relationship between the increase in the ALPS index after shunt surgery and changes in clinical rating scales. No significant correlations were found between the ALPS index increase and gait scores (TUG), total INPHGS scores, or sub‐item scores, possibly due to a small sample size. However, a significant positive correlation was observed between the postoperative rise in the ALPS index and cognitive improvement, suggesting enhanced glymphatic function may aid cognitive recovery in iNPH patients. The ALPS index selects ROIs in projection and association fiber regions at the lateral ventricle level, avoiding primary diffusion directions (y/z‐axis) to capture perivascular space (x‐axis) water mobility, thereby indirectly reflecting glymphoid function. Studies utilizing intrathecal contrast‐enhanced MRI have demonstrated that reduced ALPS index correlates with impaired global glymphatic clearance [50]. We speculated that the improvement in postoperative cognitive performance in iNPH patients may result from improved glymphatic transport function, thereby enhancing the removal of accumulated neurotoxic metabolites such as amyloid‐βand phosphorylated tau. PET studies demonstrate that iNPH patients with more severe preoperative amyloid deposition exhibit greater cognitive gains post‐shunt [51], suggesting that glymphatic reactivation may preferentially benefit those with preexisting Aβburden. Cross‐sectional and longitudinal studies across neurodegenerative diseases (e.g., AD, CSVD) consistently link lower ALPS indices to elevated Aβ/tau deposition and accelerated cognitive decline [52, 53, 54]. In iNPH, the correlation between ALPS index elevation and MMSE improvement aligns with this paradigm, positioning glymphatic restoration as a shared mechanism for cognitive recovery [55]. Rodent models further corroborate the role of glymphatic enhancement in cognitive rescue: Voluntary exercise in aged mice promotes Aβ clearance via glymphatic activation, attenuating neuroinflammation and preserving spatial memory [25]. Polyunsaturated fatty acid supplementation rescues depression‐related cognitive deficits by restoring glymphatic flow and cerebrovascular integrity in rodents [26]. These findings underscore glymphatic augmentation mitigates neurotoxicity and supports cognitive function. In addition, the pathophysiology of iNPH likely involves a cascade of CSF dynamic disturbances: early ventricular expansion induces mechanical compression of PVS surrounding perforating arteries, compromising structural integrity through reduced cross‐sectional area and diminished pulsation transmission efficiency. A seminal study in iNPH patients demonstrated that preoperative ALPS index reductions correlate with elevated CSF pulse pressure and abnormal DTI metrics (axial diffusivity, radial diffusivity), further linking glymphatic dysfunction to both hydrodynamic disturbances and microstructural damage [56]. CSF diversion surgery appears to reverse this process through decompression‐mediated mechanisms—enhancing arterial pulsatility, restoring PVS functionality, and reestablishing metabolic waste clearance capacity. Therefore, the glymphatic system potentially explains the variable cognitive outcomes observed in iNPH patients following shunt procedures. These findings offer new insights into treating cognitive impairment by enhancing brain lymphatic function, particularly for patients who are not suitable candidates for shunt surgery.

This study has several limitations: (i) The sample only includes iNPH patients who responded positively to shunt surgery. Expanding the sample to include non‐responders and those with other cognitive disorders is crucial to better understand glymphatic function differences and prevent unnecessary shunt surgeries in non‐iNPH patients. (ii) Multiple dimensions of cognitive function may be influenced by different brain regions and neural pathways. The use of MMSE to assess cognitive function limits the exploration of how glymphatic dysfunction impacts specific cognitive subdomains. (iii) The DTI‐ALPS method is limited by subjective ROI placement, affecting its stability and reproducibility. While it measures water mobility along PVS, it doesn't directly assess glymphatic flow dynamics. Future research should focus on advanced imaging techniques, like dynamic diffusion‐weighted imaging [57]. Additionally, systematic integration with complementary biomarkers (e.g., PVS dilation, free‐water mapping, the coupling strength of global blood‐oxygen‐level‐dependent (gBOLD) signals and CSF inflow dynamics, and so on) will be critical for elucidating the glymphatic system's structural and functional impacts. (iv) Our longitudinal analysis sample was smaller than our cross‐sectional analysis, which may introduce potential bias. Future studies should include larger samples and longer follow‐up periods.

## 5 Conclusion

The brain glymphatic function of iNPH patients is impaired, but it can be improved after shunt surgery and has a positive effect on the recovery of cognitive function. Clinically, monitoring brain glymphatic function would assist in predicting and evaluating the potential effects of shunt surgery, thereby enabling accurate patient screening for those who may benefit from surgical intervention.

## Author Contributions

**Yifeng Yang:** methodology, software, conceptualization, formal analysis, visualization, writing – original draft, validation, investigation, writing – review and editing. **Meijing Yan:** data curation, writing – review and editing, validation, formal analysis, investigation. **Xiao Liu:** data curation, visualization, writing – review and editing, investigation, validation. **Shihong Li:** conceptualization, writing – review and editing, supervision, methodology, validation, project administration. **Guangwu Lin:** conceptualization, methodology, funding acquisition, writing – review and editing, project administration.

## Conflicts of Interest

The authors declare no conflicts of interest.

## Supporting information


Appendix S1.

## Data Availability

The data that support the findings of this study are available from the corresponding authors upon reasonable request.
