# Supplementary material for: Alterations of Glymphatic System Before and After Shunt Surgery in Patients With Idiopathic Normal Pressure Hydrocephalus: A Longitudinal Study
Source: Eur J Neurol. 2025 May 14;32(5):e70200. doi: 10.1111/ene.70200 (PMC12076058; doi:10.1111/ene.70200)
Supplement: Supplementary file 1 — Appendix S1. [file ENE-32-e70200-s001.docx]

**Supplementary Materials**

**Inclusion and exclusion criteria and results for participants**

Patients with iNPH were diagnosed according to the second edition of the Japanese iNPH guidelines [1] for those enrolled before 2021, and according to the third edition [2] for those enrolled thereafter. To ensure compatibility with the updated imaging criteria, all MRI studies conducted prior to 2021 underwent a retrospective evaluation for features of disproportionately enlarged subarachnoid-space hydrocephalus (DESH). The diagnostic criteria for iNPH were as follows: (1) age over 60 years; (2) the presence of at least one of the classic triad symptoms (gait disturbance, dementia, and urinary incontinence) with insidious progression lasting more than six months; (3) ventricular enlargement (Evans’ index ≥ 0.3) and the presence of DESH on MRI; (4) cerebrospinal fluid pressure of 200 mm H2O; (5) absence of other diseases that could account for these symptoms; (6) positive response to CSF tap test (≥20% improvement in gait/neuropsychological metrics) and sustained symptom alleviation post-shunt surgery. The exclusion criteria for iNPH were as follows: (1) recent heavy alcohol consumption or clear causes leading to cerebral infarction or dementia due to severe psychiatric illness requiring hospitalization; (2) secondary normal pressure hydrocephalus. The inclusion criteria for healthy elderly individuals were as follows: (1) age over 60 years; (2) absence of gait disturbances, cognitive impairment, or urinary issues, with a normal MMSE score; (3) no abnormalities detected on routine brain MRI; (4) no active neurological, systemic, or psychiatric diseases.

**MRI protocol**

All MRI examinations were performed with a 3T MRI system (MAGNETOM Prisma, Siemens AG). The parameters for T1-MPRAGE were as follows: repetition time (TR) = 1800 ms, echo time (TE) = 2.37 ms, field of view (FOV) = 250 mm×250 mm, slice thickness = 0.85 mm, number of slices = 208. Multiplanar reconstruction of T1-MPRAGE yielded coronal images perpendicular to the anterior-posterior commissure level to facilitate the measurement of the Evans index. DTI data were obtained from all participants along 10 gradient-encoding directions with b values 0 and 1,000 s/mm2, a field-of-view (FOV) of 220 mm×220 mm with 56 slices, a slice thickness of 2 mm with a 0.6 mm gap, an acquired resolution of 1.15 mm×1.15 mm×2.00 mm, a reconstructed resolution of 0.57 mm×0.57 mm×2.00 mm, an echo time (TE) of 78 ms, and a repetition time (TR) of 4,600 ms.

**Comparison of the preoperative and postoperative clinical scores**

The INPHGS, TUG, and MMSE scores exhibited significant differences pre- and post-surgery (p < 0.001) by using paired comparisons (see Supplementary Table 2), indicating an overall improvement in clinical symptoms following shunt surgery. Results from multiple linear regression models for changes of each clinical scale score as presented in Supplementary Table 3.

Supplementary Table 2. The paired comparisons of the preoperative and postoperative clinical scores

|  | Pre-shunt | Post-shunt | *p*-value |
| --- | --- | --- | --- |
| **INPHGs** |  |  |  |
| gait | 3 (3-3) | 2 (1.25-2) | 0.000162^***^ |
| cognition | 2 (2-3) | 1 (1-2) | 0.000183^***^ |
| urination | 2 (2-3) | 1 (0.25-1) | 0.000408^***^ |
| total | 7 (5.5-8.5) | 4 (2-5.5) | 0.000326^***^ |
| **TUG** | 20.47±4.73 | 15.87±3.09 | 0.000028^****^ |
| **MMSE** | 20.50±8.71 | 23.00±7.22 | 0.000473^***^ |

*^Values are shown as median (range).^*

*^Significant differences are marked with *p < 0.05 , **p < 0.01 ,*** p < 0.001, and **** p < 0.0001.^*

Supplementary Table 3. Multiple linear regression: the changes in postoperative clinical scale scores as outcome variables ( n = 15).

|  | Sex, β-Coefficient (*p*) | Age, β-Coefficient (*p*) | ΔALPS, β-Coefficient (*p*) | ***R^2^*** |
| --- | --- | --- | --- | --- |
| Δ**INPHGs** |  |  |  |  |
| Δgait | -0.0229 (0.7767) | 0.0011 (0.8383) | 0.3584 (0.4755) | 0.0779 |
| Δcognition | 0.0261 (0.9141) | 0.0092 (0.6277) | 0.9099 (0.5435) | 0.1408 |
| Δurination | -0.2132 (0.2811) | 0.0082 (0.4487) | 0.1962 (0.8526) | 0.1509 |
| Δtotal | -0.0953 (0.4906) | 0.0047 (0.5370) | 0.3607 (0.6332) | 0.1232 |
| Δ**TUG** | -0.0252 (0.7327) | 0.0042 (0.3943) | -0.5228 (0.2501) | 0.1575 |
| Δ**MMSE** | -0.2617 (0.1670) | **-0.033(0.0135)**^*^ | **4.74 (0.001)**^**^ | 0.6813 |

*^β-coefficient, standardized coefficient beta;^*

*^Significant differences are marked with *p < 0.05 , **p < 0.01 and *** p < 0.001.^*

References

1. Mori E, Ishikawa M, Kato T, et al. Guidelines for management of idiopathic normal pressure hydrocephalus: second edition. Neurol Med Chir (Tokyo). 2012;52(11):775-809.
2. NAKAJIMA M, YAMADA S, MIYAJIMA M, et al. Guidelines for Management of Idiopathic Normal Pressure Hydrocephalus (Third Edition): Endorsed by the Japanese Society of Normal Pressure Hydrocephalus [J]. Neurol Med Chir (Tokyo), 2021, 61(2): 63-97.
